# Microarray analysis of iron deficiency chlorosis in near-isogenic soybean lines

**DOI:** 10.1186/1471-2164-8-476

**Published:** 2007-12-21

**Authors:** Jamie A O'Rourke, Dirk V Charlson, Delkin O Gonzalez, Lila O Vodkin, Michelle A Graham, Silvia R Cianzio, Michael A Grusak, Randy C Shoemaker

**Affiliations:** 1 Department of Genetics, Developmental and Cellular Biology, Iowa State University, Ames, Iowa 50011, USA; 2 Department of Crop, Soil, and Environmental Sciences. University of Arkansas, Fayetteville, Arkansas 72704, USA; 3 Department of Crop Sciences, University of Illinois Urbana-Champaign, Urbana, Illinois 61801 USA; 4 USDA-ARS, Corn Insect and Crop Genetics Research Unit, Iowa State University, Ames, Iowa 50011, USA; 5 Agronomy Department, Iowa State University, Ames, Iowa 50011, USA; 6 USDA-ARS Children's Nutrition Research Center, Department of Pediatrics, Baylor College of Medicine, Houston, Texas 77030, USA

## Abstract

**Background:**

Iron is one of fourteen mineral elements required for proper plant growth and development of soybean (*Glycine max *L. Merr.). Soybeans grown on calcareous soils, which are prevalent in the upper Midwest of the United States, often exhibit symptoms indicative of iron deficiency chlorosis (IDC). Yield loss has a positive linear correlation with increasing severity of chlorotic symptoms. As soybean is an important agronomic crop, it is essential to understand the genetics and physiology of traits affecting plant yield. Soybean cultivars vary greatly in their ability to respond successfully to iron deficiency stress. Microarray analyses permit the identification of genes and physiological processes involved in soybean's response to iron stress.

**Results:**

RNA isolated from the roots of two near isogenic lines, which differ in iron efficiency, PI 548533 (Clark; iron efficient) and PI 547430 (IsoClark; iron inefficient), were compared on a spotted microarray slide containing 9,728 cDNAs from root specific EST libraries. A comparison of RNA transcripts isolated from plants grown under iron limiting hydroponic conditions for two weeks revealed 43 genes as differentially expressed. A single linkage clustering analysis of these 43 genes showed 57% of them possessed high sequence similarity to known stress induced genes. A control experiment comparing plants grown under adequate iron hydroponic conditions showed no differences in gene expression between the two near isogenic lines. Expression levels of a subset of the differentially expressed genes were also compared by real time reverse transcriptase PCR (RT-PCR). The RT-PCR experiments confirmed differential expression between the iron efficient and iron inefficient plants for 9 of 10 randomly chosen genes examined. To gain further insight into the iron physiological status of the plants, the root iron reductase activity was measured in both iron efficient and inefficient genotypes for plants grown under iron sufficient and iron limited conditions. Iron inefficient plants failed to respond to decreased iron availability with increased activity of Fe reductase.

**Conclusion:**

These experiments have identified genes involved in the soybean iron deficiency chlorosis response under iron deficient conditions. Single linkage cluster analysis suggests iron limited soybeans mount a general stress response as well as a specialized iron deficiency stress response. Root membrane bound reductase capacity is often correlated with iron efficiency. Under iron-limited conditions, the iron efficient plant had high root bound membrane reductase capacity while the iron inefficient plants reductase levels remained low, further limiting iron uptake through the root. Many of the genes up-regulated in the iron inefficient NIL are involved in known stress induced pathways. The most striking response of the iron inefficient genotype to iron deficiency stress was the induction of a profusion of signaling and regulatory genes, presumably in an attempt to establish and maintain cellular homeostasis. Genes were up-regulated that point toward an increased transport of molecules through membranes. Genes associated with reactive oxidative species and an ROS-defensive enzyme were also induced. The up-regulation of genes involved in DNA repair and RNA stability reflect the inhospitable cellular environment resulting from iron deficiency stress. Other genes were induced that are involved in protein and lipid catabolism; perhaps as an effort to maintain carbon flow and scavenge energy. The under-expression of a key glycolitic gene may result in the iron-inefficient genotype being energetically challenged to maintain a stable cellular environment. These experiments have identified candidate genes and processes for further experimentation to increase our understanding of soybeans' response to iron deficiency stress.

## Background

The ability of iron (Fe) to serve as an electron acceptor makes Fe a valuable cofactor in a variety of plant processes including photosynthesis, respiration, and seed development. In the soil matrix, Fe exists in one of two forms, Fe^2+^or Fe^3+^. However, many environmental conditions, including the high pH of calcareous soils, can result in little Fe^2+ ^availability [1-4]. To survive in iron limiting environments, plants have evolved two iron uptake strategies, Strategy I and II [5]. Dicot species, including soybean, utilize the Strategy I mechanism to take up the Fe^2+ ^ion. Strategy I plants utilize an ATPase to secrete protons from the roots to acidify the rhizosphere [1,6-9]. This acidification aids in the release of Fe from chelating agents in the soil. A root membrane reductase reduces the prevalent Fe^3+^ion to the biologically usable Fe^2+ ^ion. This Fe^2+ ^can then be transported into the roots of the plant where it is available for use in various cellular processes. For strategy I plants, the iron reduction by plant roots has been identified as the rate-limiting step in iron deficiency [10]. Strategy II plants, monocot species, release phytosiderophores from the roots that chelate Fe^3+ ^ions. The entire phytosiderophore iron complex is then transported into the root system of the plant.

Complex genetic and environmental interactions have made soybean IDC an extremely difficult trait to study in field trials [11,12]. Low Fe availability exacerbates chlorosis levels in many cultivars. This is true in the calcareous soils prevalent in the upper U.S. Midwest farmlands [12]. As plants are subjected to Fe deficiency stress, they respond in a characteristic manner. Developing trifoliates exhibit interveinal chlorosis, growth is stunted, and yield is reduced. Yield reduction has a positive linear correlation with increasing chlorosis levels [4]. To minimize the environmental effect on the plant phenotype, visual phenotypic studies have been conducted with plants grown in a nutrient solution hydroponics system. The hydroponics experiments identified the same QTLs identified in field grown trials [13] making this a viable system in which to study the effects of IDC on soybean while minimizing environmental effects. The comparison of expression profiles, via utilization of cDNA microarrays, of RNA from Fe efficient and inefficient soybean near isogenic lines (NILs) grown under Fe limited hydroponic conditions will identify differentially expressed transcripts related to iron stress. This will provide clues to the physiological differences between iron efficient and inefficient cultivars

## Results

Transcript levels of near isogenic soybeans, Clark (Fe-efficient) (PI 548533) and IsoClark (Fe-inefficient) (PI 547430) were compared by microarray analysis. Plants were grown in Fe limited (50 uM Fe(NO_3_)_3_) hydroponic conditions for two weeks. RNA extracted from root tissue of both Fe efficient and Fe inefficient plants was fluorescently labeled and hybridized to soybean cDNA microarray slides, containing 9,728 cDNAs representing unigene libraries Gm-r1021 and Gm-r1083 [14], in a balanced dye swap design. A comparison of three biological replicates, each with two technical replicates for a total of six hybridizations, identified 43 genes whose expression levels exceeded a two-fold difference (Tables 1 and 2). Forty-two of the forty-three identified genes were over-expressed in the Fe inefficient line in comparison to the Fe efficient genotype, while a single gene was under-expressed.

**Table 1:** Genes differentially expressed between iron near-isogenic lines that cluster with other stress induced genes.

Clone ID	Federated Ratio	P Value	Associated TIGR TC	UNIREF 100	TBLASTX UniRef DB Annotation	Cluster Members	UniRef Blast E-Value
Gm-c1004-1674	0.296	0.2128	GmTC206003	Q9SJQ9	Fructose-Bisphosphate Aldoslase	1Fe, 1PS, 1ST	1.00E-177
Gm-c1028-6047	2.176	0.0012	GmTC220166	Q9M590	Serine/Threonine Protein Kinase	3Fe, 118PS, 107ST	1.00E-103
Gm-c1028-8683	2.204	0.0417	GmTC223013	Q9XHP4	Peroxissomal Copper Containing Oxidase	1Fe, 2PS, 1ST	1.00E-118
Gm-c1028-8247	2.409	0.0391	GmTC225799	Q8H0T8	Initiation Factor eIF-4 gamma	3Fe, 118PS, 107ST	0
Gm-c1004-8188	2.412	0.0366	GmTC224861	Q9XEE6	Zinc Finger Protein, Cys3His	1Fe, 3ST	1.00E-164
Gm-c1028-6637	2.536	0.1137	GmTC219139	Q9ZT44	Zinc Finger Protein, H2	2Fe, 6PS, 11ST	1.00E-55
Gm-c1028-5360	2.701	0.0036	AW831928	Q9C9T6	Zinc Finger Protein	2Fe, 6PS, 11ST	2.00E-19
Gm-c1009-2360	2.597	0.0262	GmTC208403	Q9LDA7	Phosphatase type 2C/	1Fe, 5PS, 6ST/1Fe, 9PS, 5ST	1.00E-107
Gm-c1004-7092	2.639	0.0208	GmTC225579	Q56E95	Ethylene Responsive Transcription Factor	2Fe, 8PS, 33ST	8e-36
Gm-c1009-2900	2.936	0.0078	GmTC214121	Q9FE67	Ethylene Responsive Transcription Factor/Ubiquitin	2Fe, 8PS, 33ST/2Fe	1.00E-24/2.00E-64
Gm-c1028-6890	5.219	0.0163	GmTC214518	Q49976	Ubiquitin	2Fe	2.00E-63
Gm-c1028-8604	2.821	0.0935	GmTC228370	Q9AXD7	Response Regulator Protein (ARR)	1Fe, 1PS, 7ST	1.00E-49
Gm-c1028-8161	2.827	0.0246	GmTC205220	Q69IX0	RER1A	1Fe, 1PS	1.00E-67
Gm-c1028-6556	2.891	0.0340	GmTC228039	Q75HJ3	Chaperonin Protein	1Fe, 1PS	1.00E-136
Gm-c1013-3137	3.01	0.0083	GmTC228924	Q6J4N8	RuBisCo Activase Protein	1Fe, 1PS, 1ST	2.00E-95
Gm-c1013-2333	3.117	0.0026	GmTC209508	Q2V2S5	SNARE Protein	2Fe, 31PS, 15ST	1.00E-143
Gm-c1028-1706	3.138	0.0091	GmTC230619	Q9SKM5	RNA Methyltransferase	1Fe, 1PS	2.00E-47
Gm-c1028-2326	3.156	0.0361	AW704123	Q9ZNZ6	Peroxidase Precursor	1Fe, 22PS, 5ST	1.00E-26
Gm-c1028-1633	3.214	0.0713	GmTC218842	Q9SPJ5	Dihydroflavonol 4 Reductase	1Fe, 4PS, 3ST	2.00E-66
Gm-c1028-5349	3.583	0.0018	GmTC204156	Q9M6R1	Heat Shock Protein Hsp70	1Fe, 2ST/1Fe, 2PS, 1ST	1.00E-115
Gm-c1004-6630	3.593	0.1557	GmTC206397	O80567	RNA Binding Protein	1Fe, 1PS, 1ST	1.00E-48
Gm-c1028-2676	3.776	0.0542	GmC225028	Q946J9	Aquaporin Protein PIP1	2Fe, 31PS, 15ST	1.00E-153
Gm-c1028-4123	5.576	0.027	AW666293	No UniRef	No UniRef Hit E < 10E-4	1Fe, 2PS, 2ST	N/A
Gm-c1028-9215	5.174	0.1109	GmTC216364	Q9MA17	Map Protein Kinase	3Fe, 118PS, 107ST	0

The clone ID identifies the specific clone spotted on the microarray. The Federated Ratio is the fold change between the two near isogenic lines. Fold changes above 2 represent genes over-expressed in the iron inefficient plant compared to the iron efficient plant while fold changes below 0.5 represent genes under-expressed in the iron inefficient plant compared to the iron efficient plant. The TIGR TC represents the tentative consensus sequence to which the clone ID belongs according to TIGR. The UNIPROT annotation is the identified function of genes showing high similarity to the sequence of the TIGR TC. Cluster members indicate the number of genes induced by iron deficiency (Fe), phosphorus depravation (PS), or general abiotic stress (ST) that share high sequence similarity to form a unique cluster grouping. The E-value is the association of the annotation to the TIGR TC sequence.

**Table 2:** Genes differentially expressed between near-isogenic lines that did not cluster with other iron or stress induced genes.

Clone ID	Federated Ratio	P-Value	Associated TIGR TC	UniProt	TBLASTX UniProt DB Annotation	UniProt Blast E-Value
Gm-c1028-8390	2.217	0.1037	BE021708	Q7XZ14	Transcription Factor DP1	7.00E-11
Gm-c1028-6580	2.247	0.0403	GmTC215393	Q06364	26S Proteasome non-ATPase Regulatory Subunit	1.00E-177
Gm-c1028-4867	2.379	0.0316	AW831377	Q8RWY1	2OG-Fe(II) Oxygenase	1.00E-25
Gm-c1028-7485	2.471	0.0311	GmTC219105	Q940G0	Endomembrane Protein	1.00E-133
Gm-c1028-2190	2.471	0.0426	GmTC227948	Q6DBF6	Membrane Protein	3.00E-35
Gm-c1028-720	2.663	0.0065	GmTC227091	Q949M9	Putative arsA Homolog hASNA-1	1.00E-147
Gm-c1004-5020	2.772	0.0241	GmTC225133	Q8JUF1	Large Polyprotein 2	0
Gm-c1004-6717	2.788	0.1011	GmTC203969	Q7XYW5	Plant Specific Membrane Protein	3.00E-18
Gm-c1009-2578	2.892	0.0551	AW278268	No UniRef	No UniRef Hit E < 10^-4^	NA
Gm-c1028-8336	3.087	0.0004	BE021665	No UniRef	No UniRef Hit E < 10^-4^	NA
Gm-c1004-6231	3.429	0.0599	GmTC204328	Q3HVN0	Ubiquitin Conjugating Enzyme	5.00E-59
Gm-c1013-2943	3.435	0.1685	GmTC226909	Q9C9T6	Zinc Ring Finger Protein	8.00E-61
Gm-c1028-1850	3.532	0.0179	GmTC229698	Motif Analysis	TIR-NBS-LRR-TIR Type Disease Resistance Protein	NA
Gm-c1028-4530	3.532	0.0102	AW704680	Q1SL19	Nonsense Mediated Decay Protein UPF3	3.00E-51
Gm-c1028-8658	3.564	0.0041	BE021924	Q6W5B6	Ethylene Receptor	4.00E-11
Gm-c1028-8183	3.687	0.2337	BE021484	Q9LR39	No UniRef Hit E < 10E-4	NA
Gm-c1028-3740	3.712	0.0359	GmTC217970	Q9LY38	Phagocytosis and Cell Motility Protein	3.00E-32
Gm-c1028-1088	4.181	0.1264	GmTC217285	Q8H9B4	UDP-glucosyltransferase	1.00E-179
Gm-c1028-963	7.149	0.0057	GmTC225698	Q2TE73	Zinc Ring Finger Protein	1.00E-108

This list of genes represents the genes identified as differentially expressed between the NILs under iron deficient conditions which do not have sequence homology to other known stress induced genes. The sequences of these genes also showed no homology to other genes induced by iron deficiency and differentially expressed between the NILs. The clone ID identifies the specific clone spotted on the microarray. The Federated Ratio is the fold change between the two near isogenic lines. Fold changes above 2 represent genes over-expressed in the iron inefficient plant compared to the iron efficient plant while fold changes below 0.5 represent genes under-expressed in the iron inefficient plant compared to the iron efficient plant. The TIGR TC represents the tentative consensus sequence to which the clone ID belongs according to TIGR. The UNIPROT annotation is the identified function of genes showing high similarity to the sequence of the TIGR TCThe E-value is the association of the annotation to the TIGR TC sequence.

As controls, the NILs were also grown in Fe sufficient hydroponics solutions (100 uM Fe(NO_3_)_3_) and analyzed on cDNA arrays containing the original 9,728 genes from root specific cDNA libraries examined plus an additional 9,272 genes from seed coat, seedling, cotyledon, flower, and pod cDNA libraries for a more global transcript analysis. An analysis of three biological replicates, with two technical replicates apiece for a total of six hybridizations, showed no genes with consistent differential expression between the NILs under Fe sufficient conditions. Thus, the differential expression seen under Fe deficient conditions is likely a result of the differential response of the NILs to the Fe limited environment rather than inherent genetic differences between the NILs [15].

Real Time RT-PCR experiments confirmed the expression patterns observed in the microarray experiments for nine out of ten randomly chosen genes (Table 3 with figures in supplemental data: [16]). These experiments confirmed that, for the genes tested, the Fe inefficient plants had higher levels of gene expression than Fe efficient plants (Table 3 and[16]). For four of the nine genes confirmed, the RT-PCR results showed greater differential expression between the NILs than was identified by microarray analysis. The RT-PCR experiments examined expression patterns of individual genes, as evidenced by the single peak in the melting curve analysis (data not shown), while hybridization-based microarrays do not necessarily distinguish between gene family members. Three of the nine genes examined by RT-PCR clustered with known stress response genes while the other six genes analyzed by RT-PCR appear to be unique to soybean's iron deficiency response (see below).

**Table 3:** Real Time PCR results confirming differential expression identified by microarray analysis

Clone ID	Forward Primer	Reverse Primer	Federated Ratio	Fold Change Identified by Real Time RT PCR	Fe Efficient Standard Error	Fe Inefficient Standard Error
Gm-c1028-4867	CAGTGGAACTTCGTTGGG	AAAAGGCCTGGAATGCTC	2.379	7.56	0.345	0.255
Gm-c1004-8188	CCCTGATCTAGAAGTTGG	GCAGGAGCAGATGGTAGC	2.412	2.9	0.185	0.015
Gm-c1028-5360	CAGTGGAACTTCGTTGGG	AAAAGGCCTGGAATGCTC	2.701	2.7	0.115	0.030
Gm-c1004-5020	GAAGAACAGCGAAACCTAAC	CGGCTACTCCCTATCCA	2.772	2.7	0.020	0.040
Gm-c1028-2326	CAAGAGCATGATCTACCAGC	GGACAGAGGGAGAGATCAGG	3.156	2.82	0.080	0.040
Gm-c1013-2943	CGAACCCAAACAAGATACAC	GATTGTATTTCCCGTGGATT	3.453	5.12	0.040	0.060
Gm-c1028-8658	TCCAACTCCATCGTCGAG	GTGAATGCGCGAAGGAT	3.564	4.2	0.055	0.010
Gm-c1028-8183	CCAAGCTGGACCATA	ACATTGGCTATTTACTTACA	3.687	3.66	0.025	0.045
Gm-c1028-963	TGCCATCACTGTTTATCAAG	GCCACTGCCCTGTCTTACTC	7.149	2.8	0.060	0.05

Ten genes were chosen at random to have their differential expression between Clark (Fe-efficient) and IsoClark (Fe-inefficient) identified by microarray analysis confirmed by semi-quantitative real time PCR analysis. Differential expression was confirmed for nine of the ten genes chosen. In four of the nine genes the real time PCR showed greater differential expression between the NILs than was identified by microarray analysis.

To determine a probable function of the 43 differentially expressed genes, the GenBank accession of the Expressed Sequence Tag (EST) for the gene was queried against The Institute for Genomic Research (TIGR) database soybean gene index (Version 12.0) [17] to identify the tentative consensus (TC) sequence containing the respective EST. The TC sequence was compared to the UniProt protein database (February 2006) [18] using BLASTX [19] and an E-value cutoff of E < 10^-4^, to assign a putative function (Tables 1 and 2). Eight of the forty-three sequences examined had no homology to the UniProt protein database. Therefore, the eight individual EST sequences (Gm-c1028-8183, Gm-c1028-8336, Gm-c1009-2578, Gm-c1028-4530, Gm-c1028-1850, Gm-c1028-5360, Gm-c1028-963, and Gm-c1028-4123) were queried against a database of available Soybean Whole Genome Shotgun (WGS) using megaBLAST BLASTN with an E-value cutoff of E < 10^-100 ^to identify genomic sequence that could extend the EST sequence. Identical sequence reads which were at least 500 nucleotides in length and shared 100% nucleotide identity to the EST were assembled into a multiple sequence alignment with the EST. If any of the identified sequences extended the ends of the EST a new consensus was generated for the EST. The new consensus was then compared to the UniProt database by BLASTX with an E-value cutoff of E < 10^-4 ^to assign a putative function.

Genes known to be involved in the Fe deficiency response have been identified and characterized in model organisms such as *Arabidopsis thaliana*. To determine if homologs of these genes were present on the soybean cDNA array, 33 members of six Arabidopsis gene families known to be involved in Fe uptake and homeostasis (IRT, FRO, FRD, FIT, NRAMP and YSL) were compared to the soybean EST database by BLASTN comparison (E < 10^-4^). Soybean EST sequences belonging to the Gm-r1021 and Gm-r1083 libraries, and thus putatively represented on the cDNA array, were identified. The soybean sequences were then compared (BLASTN) back to the Arabidopsis genome to determine if they were the reciprocal best match to the original Arabidopsis iron genes and likely functional orthologs. This approach demonstrated that only one soybean ortholog of an Arabidopsis iron uptake gene was represented on the array. Soybean EST Gm-r1083-2131 is the homolog of Arabidopsis Yellow Stripe-Like 6. However, this gene was not differentially expressed in the microarray experiment.

A single linkage cluster analysis [20] was performed to identify any Fe induced genes with sequence homology (E < 10^-4^) to other stress induced genes. Twenty-four of the 43 Fe deficiency induced genes clustered with known stress-induced genes (Table 1). Most clusters contain only one Fe induced gene and a number of other stress induced genes. However, one cluster was composed of only two genes (Gm-c1009-2900 and Gm-c1028-6890) which showed homology to each other and were differentially expressed under Fe deficient conditions, but show no significant homology to other stress induced genes. The remaining nineteen Fe deficiency induced genes showed no sequence homology to known stress induced genes, nor to the other Fe deficiency induced genes identified by the microarray experiment (Table 2).

**Figure 1:**
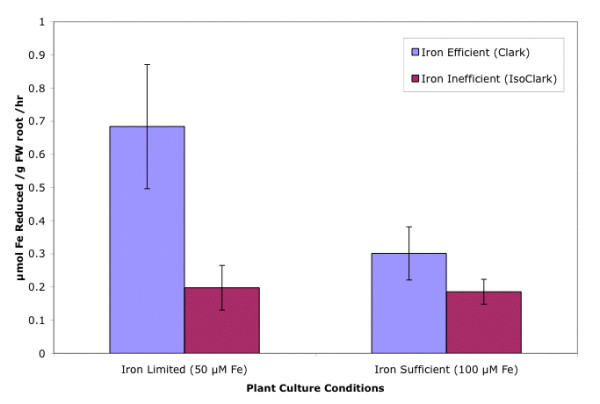
**Whole Root Reductase Assay Results Across Various Iron Concentration Growth Conditions**. The iron efficient Clark plant shows a statistically significant increase in reductase activity at 50 uM Fe(NO3)3, iron deficient conditions for the microarray experiment. At the same iron concentration, the iron inefficient IsoClark shows low levels of reductase activity.

Because iron reductase is a fundamental component of Strategy I plants, but not represented on the cDNA array, we conducted root iron reductase experiments on both iron efficient and inefficient plants grown in hydroponic solutions 50 and 100 uM Fe(NO_3_)_3_. This provided us with information on the physiological status of the plants for this enzyme activity. The iron efficient plant showed a statistically significant increase in root reductase activity from 0.2 to 0.7 umol Fe reduced per gram of fresh weight tissue per hour at 50 uM Fe(NO_3_)_3 _(Figure 1).

## Discussion

In calcareous soils iron-inefficient soybean genotypes often display symptoms of iron deficiency stress (interveinal chlorosis and reduced yield). These symptoms are exacerbated by the cool wet conditions prevalent in early spring. Under field conditions, if the young soybean plant survives the initial iron stress, the plant continues to grow, albeit slowly, and eventually, as the plant matures and the environmental conditions change, the phenotypic effects of iron stress disappear [21]. Soybean cultivars differ in their ability to respond successfully to iron stress. Results of this study have provided clues to understand some of the physiological differences between iron-efficient cultivars and iron-inefficient cultivars.

In this study, NILs developed especially for their iron deficiency response by the USDA [22], were used in an established hydroponics system to compare gene expression profiles between iron efficient (Clark) and iron inefficient (IsoClark) NILs. The NILs are phenotypically identical, except in their chlorotic response under iron stress conditions. Clark remains a healthy green under iron deficient conditions while IsoClark exhibits severe interveinal chlorosis.

Growing the NILs in an established hydroponics system allowed for a comparison of gene expression profiles of the roots of iron efficient (Clark) and inefficient (IsoClark) plants to identify differentially expressed genes between the NILs to better understand the physiological responses of soybean to iron stress. Most changes in gene expression identified under iron-limited conditions are negated upon the re-supply of iron to the system [15], thus confirming these genes as induced by iron deficiency. This comparison allows us to confidently report these forty-three genes as differentially expressed between the NILs in response to iron deficiency.

### Non-Induction of Iron Reductase Activity Under Iron Deficiency Stress in the Iron-Inefficient Soybean Isoline

Induction of the Fe(III) chelate reductase is a key iron stress response in Strategy I plants [23]. Without reductase activity, the available Fe^+2 ^for uptake of iron into the root is extremely limited. Because the gene encoding iron reductase (FRO2) was not found on the cDNA array used in this study, we conducted an iron reductase assay to assess this uniquely-Strategy I response in both the iron efficient and inefficient genotypes. The iron inefficient genotype used in this study failed to respond to iron deficiency stress by induction of increased ferric reductase activity. However, the iron-efficient genotype responded to reduced iron availability by increasing its ability to reduce Fe^+3 ^to the usable Fe^+2^. Reduction of ferric iron by the roots is considered to be a limiting factor in successful response to reduced iron availability [10]. The lack of induction of the iron reductase in the inefficient isoline is likely a major factor contributing to the severe iron deficiency stress symptoms observed, relative to the iron efficient genotype.

### General and Specialized Stress Response Genes are Involved in Soybean Iron Deficiency Stress Response

With the advent of microarray technology, researchers can now identify a broad range of genes that work in concert to protect the plant from abiotic and biotic stresses. While some genes may be specific to a particular pathogen, stress, or plant species, others may be part of a general stress response shared across multiple plant species or multiple stresses. We developed an in-house sequence database that contains genes identified from the literature that are significantly differentially regulated in response to abiotic or biotic stresses. Some of the sequences are differentially expressed in response to pathogen attack [24,25] while the majority, are differentially expressed in response to a variety of abiotic stresses including oxygen deprivation [26], drought [27], salt stress [28,29], Fe deficiency [30], oxidative stress [31], phosphate deficiency and others [32-34]. Included in this database are the 43 genes identified in our experiments as differentially expressed in response to limited Fe.

A single linkage cluster analysis [20] was performed to determine if the genes identified as differentially expressed in our microarray experiment exhibited significant (10E^-4^) sequence similarity to genes differentially expressed under other abiotic stress conditions. Twenty-four of the 43 identified genes showed significant sequence similarity to other genes whose expression levels are altered by some form of abiotic stress (Table 1). The remaining 19 genes showed no sequence homology to known stress induced genes (Table 2). These 19 genes may be unique to soybeans' iron response. Three sequences show no sequence homology to any of the genes characterized in the UniProt database, or to other genes identified under iron limited conditions. The unique sequence of these three genes suggests they may be unique to legumes. The two groupings of genes (Tables 1 and 2) identified under Fe limited conditions suggest both a universal stress response and an Fe specific stress response are induced upon Fe deficient conditions.

### The Soybean Response to Iron Deficiency Stress

Plants respond to iron stress through an impressive number of metabolic adaptations and adjustments. The iron-inefficient soybean isoline used in this study failed to respond to reduced iron availability by increased activity of Fe(III) chelate reductase. Thus, the reduced availability of the iron in the growth medium created a severe iron stress for the inefficient plants.

The most striking response of the inefficient isoline to iron stress was the dramatic increase in transcripts of genes involved in signaling and hormonal regulation. Increased signaling is likely an attempt on the part of the stressed plant to maintain metabolic homeostasis in a decreasingly sustainable environment. For example, MAP kinase and a SNARE protein are well known signaling proteins that were induced in the inefficient line. In addition, RNA mediating genes for RNA methyltransferase and an RNA binding protein were also induced upon iron stress, as were several DNA-binding zinc finger protein genes and ethylene receptors.

Ethylene is a signaling molecule often associated with root hair development, pathogen infection, wounding and other abiotic stresses [35] including iron stress [36-38]. In this study, transcripts encoding an ethylene receptor protein and two ethylene responsive transcription factors were up-regulated. These transcription factors are one of the largest families of transcription factors and can be induced by abiotic stresses [39]. A MAP kinase protein was also induced by iron stress in this study and has previously been shown to serve as a relay in the ethylene-signaling pathway [40]. Another up-regulated gene, the response regulator protein, has been shown to be involved in ethylene and other hormone signaling [41]. The identification of so many genes (>16% of all transcripts identified) encoding ethylene response-protein gene transcripts under our experimental conditions strongly indicates the ethylene signaling pathway is involved in the soybean Fe deficiency stress response, probably serving a myriad of duties [42].

The increase of signaling transcripts in the severely stressed genotype likely accounts for the up-regulation of genes involved in anion transport (endomembrane protein and the putative arsA Homolog hASNA-1) and an aquaporin protein. Aquaporins are known to be induced by iron deficiency and other abiotic stresses [43]. The induction of an aquaporin gene points toward the necessity for the cells to move nutrients and metabolites. Because of the ability of aquaporins to transport small molecules they may also be serving in the movement of additional cellular signals in stress pathways.

UDP-glucosyltransferase calalyzes the transfer of a glucosyl group from UDP-glucose to an acceptor molecule. UDP-glucosyltransferase has recently been shown to be a key enzyme in the production of isoflavones in *Glycine max *[44]. Because of the role of isoflavones in the soybean stress response the induction of this gene may simply reflect a generalized response to the iron stress. However, the induction of this gene may be indicative of glucosylation of proteins for export through cellular membranes, or synthesis of oligosaccharides from cellular starch or sugars [45]. Glucosylation of protein-linked oligosaccharides may protect them from degradation [46]. Because the endoplasmic reticulum is the main site at which glucosylation of oligosaccharides takes place, induction of an RER1-like gene (functions in returning membrane proteins to the endoplasmic reticulum (ER)) supports this scenario.

A single aldolase gene, Fructose-bisphosphate aldolase, was under-expressed in the inefficient genotype relative to the efficient genotype. The uniqueness of this response under our experimental conditions warrants discussion. The reduced amount of this catalytic gene product may have several outcomes. Fructose-bisphosphate aldolase is an early step in the glycolysis pathway. The products of this pathway are ATP and pyruvic acid (PVA). It is unlikely that suppression of this gene during severe iron stress and chlorosis means the inefficient isoline has an adequate energy source from photosynthesis and therefore does not require the breakdown of glucose. The possible slowdown of glycolysis could result in an energetically challenged cellular environment, thus contributing further to the iron stress. The lack of evidence for increased glycolysis would also suggest that glucose levels are not depleted, leaving that molecule available for other activities (see above).

Although less supported, under-expression of the aldolase gene may result in failure to induce a critical iron homeostasis response in the inefficient genotype. The reduced amount of aldolase transcript in the inefficient genotype suggests this may not have been adequate to respond to reduced iron in the environment. Therefore, in addition to the lack of ferric reductase induction, maintenance of iron homeostasis within the cell may have been further impaired, compounding the iron stress placed on the plant. This has important implications for understanding genetic variation in soybeans' response to iron stress.

These two results (lack of induction of ferric chelate reductase and reduced aldolase transcript) probably play major roles in creating the severe stress seen in the inefficient genotype. Most of the other differentially expressed transcripts can be explained by the soybean physiological responses to the stress.

Under adverse environment conditions, such as iron stress, plants are known to produce reactive oxygen species (ROS). In this study, a peroxisomal copper containing oxidase was up-regulated in the inefficient genotype. Peroxisomal copper containing oxidase catalyzes the oxidation of amines to aldehyde, NH_3 _and H_2_O_2 _[47]. ROS such as H_2_O_2 _can cause damage to proteins and lipids [48]. The hydroponic conditions maintaining severe iron deficiency stress invoke the oxidative stress response. In a seemingly defensive reaction, the up-regulation of a peroxidase precursor points to the soybean plant responding to the increased ROS (H_2_O_2_) by increasing the amount of ROS-scavenging enzyme(s). This is not unusual. Other Strategy I plants, such as sunflower and sugar beet, also have been shown to respond to iron stress through changes in components of their antioxidative systems [49,50].

Several of the up-regulated genes in iron stressed roots identified in this study are related to the ubiquitin/proteosome degradation pathway. These include ubiquitin, ubiquitin conjugating enzyme, and a 26S proteasoeme regulatory subunit. The up-regulation of genes in the ubiquitin/proteasome pathway plus the up-regulation of a gene for phagocytosis and a cell motility protein suggests a breakdown of cellular membranes and general deterioration of cellular health of root tissue due to iron deficiency.

Nutrient deprivation in plants has shown to induce both ubiquitin/proteasome and vacuolar degradation of proteins and lipids [43,51]. Homologs of these genes in other species have been shown to be involved in recycling non-essential proteins and the utilization of the degraded products to maintain vital cellular function [52]. Ubiquitin conjugating enzymes have been shown to be induced under stress conditions [43] including heavy metal stress. The ubiquitin response has also been associated with the regulation and downstream signaling of resistance genes [53]. The by products of this catabolism are thought to be re-mobilized to sustain growth under stress conditions [51]. The remobilization of the byproducts by the iron inefficient plants may provide carbon and nutrients to rapidly expanding leaves. Thimm *et al.*[54] suggested a similar physiological response to iron stress, to maintain carbon flow. Garbarino *et al.*[55] suggested abiotic stress results in improperly folded proteins, which are targeted for degradation by ubiquitinization. Interestingly, one of the over-expressed genes in the inefficient genotype encoded a chaperonin protein and chaperonins are needed for proper folding of nascent proteins.

The transcript for the ubiquitin conjugating enzyme was shown to be up regulated in iron inefficient plants under iron limiting conditions. In other species this enzyme has been shown to require the interaction of zinc ring finger proteins. In this study, five zinc finger protein genes were induced in the iron-stressed genotype. These zinc finger proteins may be acting as transcription factors in the regulation of the ubiquitin pathway in soybean, or they may be involved in the post translational modification of other genes known to be involved in iron homeostasis [56-58].

It is unlikely that protein and lipids are the only cellular components modified by the physiological conditions created from the iron stress. The increased expression of the 2-oxoglutarate (2OG) Fe(II)-dependent oxygenase suggest that the physiological changes brought about by iron deficiency stress has resulted in damage to DNA or modification of RNA and the soybean plant is responding to those challenges by increasing DNA repair and RNA stability. The 2OGFe(II)-dependent oxygenase has been predicted to detoxify methylated bases of ssDNA and reverse methylase modification of RNA, thus creating less toxic base derivatives, and enzymes of this family are also known to catalyze the formation of the plant hormone ethylene [59].

Many of the changes in transcript level observed in iron-stressed soybean correspond to general stress responses. For example, a TIR-NBS-LRR-TIR gene, a common motif in known resistance genes, was found to be up-regulated in the stressed iron-inefficient genotype. The over-expression of this gene suggests soybean responds to iron stress in a manner akin to the way it would combat pathogenic infection. Similarly, when iron-stressed, other plants such as Arabidopsis and rice, show expression changes of genes involved in wounding, abiotic and biotic stresses [60], as well as reproduction [61,62]. These types of genes may all be members of common cascades involved in physiological stress responses.

It is important to note that four of the genes we identified in this experiment had no BLAST homology to the UniProt protein database (Tables 1 and 2). While this makes it difficult to determine their function, the fact that they are induced 2.9 to 3.5 fold, suggest they have very important roles and are worthy of further functional analyses.

## Conclusion

The use of cDNA arrays has allowed us to identify transcripts differentially expressed in soybean under Fe stress conditions. Some of the genes identified are similar to general stress response genes while others may be specific to Fe stress response in soybean. It is important to note that the genes found on the cDNA array used in this study represent only a small subset of the total genic component of soybean. As such, the genes identified as differentially expressed in this study represent only a fragmented snapshot of changes occurring in the soybean physiology in response to iron deficiency stress.

However, we have been able to confirm and extend previous knowledge of soybean's iron stress responses and draw important inferences for genetic and physiological differences between soybean iron-efficient and iron-inefficient genotypes. Relative to inefficient soybean genotypes, iron-efficient genotypes may have an increased ability to respond to reduced iron availability in the environment through efficient induction of iron reductase. Root membrane bound reductase capacity is often correlated with iron efficiency. In this study, under iron limited conditions, the iron efficient plant had high root membrane reductase capacity while the iron inefficient plants reductase levels remained low, further limiting iron uptake through the root. Additionally, iron-efficient genotypes may have an efficient induction of catalytic enzymes necessary to release ATP and provide a much needed energy source to help maintain homeostasis.

Many of the genes induced in the iron inefficient NIL are involved in known stress induced pathways. The most striking response of the iron inefficient genotype to iron deficiency stress was the induction of a profusion of signaling and regulatory genes in an attempt to establish and maintain cellular homeostasis. Genes were induced that point toward an increased transport of molecules through membranes. A suppression of a key catalytic gene suggests the iron-inefficient genotype may be energetically challenged to maintain a stable cellular environment.

Many of the induced genes were obviously up-regulated in response to decreasing metabolic integrity and cellular damage. Enzymes were induced that point toward production of protein and lipid-damaging reactive oxidative species and a concomitant induction of an ROS-defensive enzyme. Genes involved in DNA repair and RNA stability were induced. Other genes were induced that are involved in protein and lipid catabolism; perhaps as an effort to maintain carbon flow and scavenge energy. These experiments have identified candidate genes and processes for further experimentation to increase our understanding of soybeans' response to iron deficiency stress.

These transcripts should serve as a starting point for future research to both understand and improve iron uptake and utilization as a step in improving overall plant health. Understanding the role these gene products play in soybean Fe metabolism could help alleviate yield loss for crops grown in calcareous soils. Further manipulation of these genes could lead to higher Fe content or increased Fe bioavailability for soybeans and other Strategy I food crops.

## Methods

Near isogenic soybean lines (NILs) were developed by the USDA in 1972 [22] specifically for their response to Fe deficiency. Fe efficient PI 548533 (Clark) was crossed with Fe inefficient PI 54619 (T203). Progeny were selfed and resulting F2 plants were screened for Fe inefficiency. The Fe inefficient progeny were backcrossed to the Fe efficient PI 548533 for six generations [22], resulting in an Fe inefficient plant with the Clark genetic background. The Fe inefficient isoline was released as PI 547430 (IsoClark) [22]. Both the Fe efficient PI 548533 (Clark) and Fe inefficient PI 547430 (IsoClark) lines were grown in the Ames, Iowa USDA greenhouse under 16 hr photoperiods. Plants were germinated in sterile vermiculite with distilled deionized water. After one week they were transplanted into a DTPA nutrient buffered hydroponics system [3] containing all minerals necessary for normal growth. Experimental 10L systems to induce Fe deficiency stress had 50 uM Fe(NO_3_)_3 _Fe levels while systems for the control experiment contained 100 uM Fe(NO_3_)_3_. Additionally, each 10 L system contained 2 mM MgSO_4_*7H_2_O, 3 mM Mg(NO_3_)_2_*6H_2_O, 2.5 mM KNO_3_, 1 mM CaCl_2_*2H_2_O, 4.0 mM Ca(NO_3_)_2_*4H_2_O, 0.020 mM KH_2_PO_4_, 542.5 uM KOH, 217 uM DTPA, 1.52 uM MnCl_2_*4H_2_O, 4.6 uM ZnSO_4_*7H_2_O, 2 uM CuSO_4_*5H_2_O, 0.20 uM NaMoO_4_*2H_2_O, 1 uM CoSO_4_*7H_2_O, 1 uM NiSO_4_*6H_2_O, 10 uM H_3_BO_3_, and 20 mM HCO_3_. A pH of 7.8 was maintained by the aeration of a 3% CO_2_: air mixture. A supplemental nutrient solution containing 16 mM potassium phosphate, 0.287 mM boric acid and 355 mM ammonium nitrate was added daily to maintain proper plant nutrition. To ensure the chlorosis was due to Fe deficiency stress, A15, an Fe efficient plant, and T203, Fe inefficient plant, were included with each experimental replication. Plants were grown in the hydroponics system for two weeks, until they reached the V3 stage [63], at which point tissue was harvested for RNA extraction. This experiment was replicated three times, for three independent biological replicates, each with two technical replicates.

### RNA Extraction and Microarray Hybridizations

Total RNA from Fe deficient plants was extracted from root tissue of three biological replicates, each with two technical replicates, for a total of six slide hybridizations using a modified phenol:chloroform extraction with a lithium chloride precipitation [14]. Total RNA for control samples was extracted from root tissue following the QiagenRNeasy protocol for three biological replicates each with two technical replicates for a total of six slide hybridizations. All samples were composed of root tissue from four individual plants, all grown in the same hydroponic unit. RNA purity was determined by spectroscopic readings at A_260 _and A_280 _and by formaldehyde gel visualization. Experimental samples were further purified using the RNeasy kits from Qiagen. Purified RNA was then re-analyzed to determine purity and final concentration. Each sample yielded 180 ug of purified RNA, 90 ug of purified RNA was used for each of the dye swap pairs of cDNA slides. The cDNA array for experimental samples consisted of 9,728 total cDNAs of unigene sets Gm-r1021 and Gm-r1083 spotted onto amine coated glass slides [14] and entered as platform GPL1013 in NCBIs Gene Expression Omnibus database [64,65]. The cDNA array for the control samples consisted of the original 9,728 total cDNAs from unigene sets Gm-r1021 and Gm-r1083 plus an additional 9,272 total cDNAs of unigene set Gm-r1070 and entered as platform GPL3015 in GEO.

Purified RNA samples were split into 90 ug aliquots and concentrated to 10 uL in a Savant Speed Vac. The concentrated purified RNA and oligo dT was heated together for 10 min. 20 uL of 1 × Buffer, 10 mM DTT, 500 uM low T dNTPs, 100 uM Cy3 or Cy5 (Amersham Biosciences), and 13 u/uL SuperScriptII (Invitrogen) was added to each RNA/Oligo dT sample then placed at 42°C for 2 hours. Remaining RNA was degraded with an RnaseA/H treatment. The three biological replicates of the Fe deficient samples formed six technical replicates, the raw data has been deposited in GEO [64,65] and is accessible through GEO series accession number GSE7290. The three biological replicates of the control samples formed six technical replicates, again, the raw data has been deposited in GEO [64,65] and is accessible through GEO series accession number GSE7325. The labeled Clark (Fe-efficient) and IsoClark (Fe-inefficient) cDNA samples were mixed in a balanced dye swap design. The combined samples were purified with QIAquick PCR purification kits (Qiagen) labeled with PolyA DNA and hybridized for 18 hours at 42°C. After overnight hybridization, slides were washed (wash 1: 1 × SSC, 0.2%SDS, wash 2: 0.2 × SSC, 0.2%SDS, wash 3: 0.1 × SDS) to remove unbound cDNAs. Slides were scanned with ScanArray Express (Stratagene) and resulting images were overlaid and spots identified by the ImaGene program. An analysis program developed at the University of Illinois [14] was used to identify differentially expressed cDNAs. For our purposes, differential expression is defined as a minimum of two fold over or under expression in the cDNA of IsoClark (Fe-inefficient) relative to Clark (Fe-efficient).

### Real Time PCR Confirmation

For the RT-Real Time PCR experiments, 200 ng of RNA extracted from root tissue of plants collected over a 48-hour time course was added as initial template for each sample with Time 0 representing the time at which tissue was collected for the microarray experiment. Primers (Table 3) were designed to produce a 250 bp amplicon based on the sequences available from GenBank. Stratagene's Brilliant qRT-PCR kit was used with each 25 uL reaction assembled as described by the Stratagene instruction manual (Catalog #600532) with 2.5 uL of 50 mM MgCl_2_, and 2 uL of 50 nM Forward and Reverse primers as determined experimentally to optimize the reactions. Cycling protocols consisted of a 45 min. at 42°C for the reverse transcription, 10 min at 95°C to disable any remaining StrataScript, then 40 cycles of 30 sec at 95°C, 1 min at proper annealing temperature for each primer pair, 30 sec at 72°C. The PCR reactions were run in the Stratagene Mx3000P followed by a dissociation curve, taking a fluorescent reading at every degree between 55°C and 95°C to ensure only one PCR product was amplifying. The Stratagene analysis system established a threshold fluorescence level where amplicon fluorescence levels were statistically higher than background fluorescence; this threshold level is referred to as the Ct value, the cycle at which the samples fluorescence is above threshold. To be considered differentially expressed, the Fe efficient and Fe inefficient plants at the same time point had to differ in where they crossed the Ct by more than 1 cycle. One cycle difference in the RT-PCR experiments corresponds to the two-fold difference in gene transcripts between the NILs examined by the microarray experiment. The fold change was calculated from the differences in Ct using the 2^ΔCt ^method [20,66]. As controls, a passive reference dye was added to each sample, to ensure recorded fluorescence levels were due to SYBR green incorporation. Additionally, each sample was also run in triplicate and each sample was also normalized against tubulin amplification, see primers in Table 3, to ensure the differential expression was not due to differing amounts of initial RNA template added to each sample. As an additional negative control each sample was also run without reverse transcriptase to ensure amplification was due from RNA template.

### Single Linkage Clustering Analysis

The single linkage clustering analysis performed in this work used techniques first reported by Graham et al. [20]. In brief, the nucleotide sequences of the 43 cDNAs identified as differentially regulated under Fe chlorosis conditions were added to a data set containing the nucleotide sequences of plant genes known to be involved in general stress responses. These general stress genes were identified based on micro/macro and bioinformatic analyses from the following published works: [52,24-26,67,28,68,33,30,31,20,34]. Of the total 430 sequences used for clustering, 221 were derived from phosphate-starved tissues of Arabidopsis [34], *Medicago truncatula*, soybean and *Phaseolus vulgaris *[20]. The remaining 209 sequences came from a variety of plant stresses [52,24-28,68,33,30,31]. Each sequence was given a unique identifier to allow identification of the source treatment. The entire data set was then compared to itself using TBLASTX [19] with a minimum E-value cutoff of 10E^-4^. The single linkage clustering perl scripts generated by [20] were used to assign homologous sequences to a cluster. Note that sequences with no UniProt hit, can cluster to sequences with known annotation. Thus, clustering can be used to imply annotation.

### Root Iron Reductase Analysis

Seeds of iron efficient and inefficient plants were germinated on germination paper for 7 days before being transplanted into the hydroponics system described above, with either 50 or 100 uM Fe(NO_3_)_3_. Plants were grown for 2 weeks in the hydroponics system. Cotyledons were removed after 7 days in hydroponics to ensure a uniform chlorotic response. Root reductase activity of the plants was measured with intact roots that were submerged for 30 min in an aerated assay solution containing 1.5 mM KNO_3_, 1 mM Ca(NO_3_)_2_, 3.75 mM NH_4_H_2_PO_4_, 0.25 mM MgSO_4_, 25 uM CaCl_2_, 25 uM H_3_BO_3_, 2 uM MnSO_4_, 2 uM ZnSO_4_, 0.5 uM CuSO_4_, 0.5 uM H_2_MoO_4_, 0.1 uM NiSO_4_, 100 uM Fe(III)-HEDTA, 100 uM BPDS (bathophenanthroline disulfonic acid), and 1 mM MES, pH 6. Iron reduction was quantified spectrophotometrically, by measuring the formation of the red-colored product, Fe(II)-BPDS_3_; absorbance was measured at 535 nm. An aliquot of the solution with no roots submerged in it is used as the blank. A molar co-extinction coefficient of 22.14 mM^-1^cm^-1 ^was used with the measured absorbance reading to calculate the rate of reduction. There were two replicates of the experiment, each with three plants per genotype per iron concentration.

## Authors' contributions

JAO carried out the sample collection and preparation, microarray hybridizations, RT PCR validation, data analysis and drafted the manuscript. DVC provided advice on experimental design and provided comment and revisions to the manuscript. MAG (Ames) carried out the bioinformatic analysis, including the single linkage clustering and annotation, and assisted in drafting the manuscript. LOV provided leadership in cDNA microarrays, their analysis, and edited the manuscript. DOG produced cDNA microarrays used in the study and aided with the hybridizations. SRC provided comments and revisions to the manuscript. MAG (Houston) assisted in the reductase assays and provided comments and revisions to the manuscript. RCS conceived the study, coordinated the design of the project, and drafted the manuscript. Authors are grateful for helpful discussions with Professors R. Thornburg and J. Specht. All authors read and approved the final manuscript.

## References

[B1] Mengel K (1994). Iron Availability in Plant Tissues – Iron chlorosis on calcareous soils. Plant and Soil.

[B2] Mengel K, Kirby EA, Kosegarten H, Appel T (2001). Principles of Plant Nutrition.

[B3] Coulombe BA, Chaney RL, Wiebold WJ (1984). Bicarbonate Directly Induces Iron Chlorosis in Susceptible Soybean Cultivars. Soil Science Society American Journal.

[B4] Froelich DM, Fehr WR (1981). Agronomic Performance of Soybeans with Differing Levels of Iron Deficiency Chlorosis on Calcareous Soil. Crop Science.

[B5] Romheld V (1987). Different Strategies for Iron Acquisition in Higher Plants. Physiol Plant.

[B6] Rogers EE, Guerinot ML (2002). FRD3, a Member of the Multidrug and Toxin Efflux Family, controls Iron Deficiency Responses in Arabidopsis. The Plant cell.

[B7] Vert G, Grotz N, Dedaldechamp F, Gaymard F, Guerinot ML, Briat JF, Curie C (2002). IRT1, an Arabidopsis transporter essential for iron uptake from the soil and for plant growth. The Plant cell.

[B8] Eide D, Broderius M, Fett J, Guerinot ML (1996). A novel iron-regulated metal transporter from plants identified by functional expression in yeast. Proceedings of the National Academy of Sciences of the United States of America.

[B9] Vert G, Briat JF, Curie C (2001). Arabidopsis IRT2 gene encodes a root-periphery iron transporter. Plant J.

[B10] Connolly EL, Campbell NH, Grotz N, Prichard CL, Guerinot ML (2003). Overexpression of the FRO2 ferric chelate reductase confers tolerance to growth on low iron and uncovers posttranscriptional control. Plant Physiol.

[B11] Cianzio SR, Fehr WR, Anderson IC (1979). Genotypic Evaluation for Iron Deficiency Chlorosis in Soybeans by Visual Scores and Chlorophyll Concentration. Crop Science.

[B12] Lin S, Cianzio SR, Shoemaker RC (1997). Mapping genetic loci for iron deficiency chlorosis in soybean. Molecular Breeding.

[B13] Lin S, Baumer JS, Ivers D, Cianzio SR, Shoemaker RC (1998). Field and Nutrient Solution Tests Measure similar Mechanisms Controlling Iron Deficiency Chlorosis in Soybean. Crop Science.

[B14] Vodkin LO, Khanna A, Shealy R, Clough SJ, Gonzalez DO, Philip R, Zabala G, Thibaud-Nissen F, Sidarous M, Stromvik M, Shoop E, Schmidt C, Retzel E, Erpelding J, Shoemaker RC, Roxigues-Huete AM, Polaco JC, Coryell V, Keim P, Gong G, Liu G, Pardinas J, Schweitzer P (2004). Microarrays for global expression constructed with a low redundancy set of 27,500 sequenced cDNAs representing an array of developmental stages and physiological conditions of the soybean plant. BMC genomics.

[B15] O'Rourke JA, Graham MA, Vodkin L, Gonzalez DO, Cianzio SR, Shoemaker RC (2007). Recovering from iron deficiency chlorosis in near-isogenic soybeans: a microarray study. Plant Physiol Biochem.

[B16] Iron Deficiency Supplemental Data. http://soybase.org/publication_data/ORourke/irondeficiency/index.html.

[B17] Quackenbush J, Cho J, Lee D, Liang F, Holt I, Karamycheva S, Parvizi B, Pertea G, Sultana R, White J (2001). The TIGR Gene Indicies: analysis of gene transcript sequences in highly sampled eukaryotic species. Nucleic Acids Res.

[B18] Apweiler R, Bairoch A, Wu CH, Barker WC, Boeckmann B, Ferro S, Gasteiger E, Huang H, Lopez R, Magrane M, Martin MJ, Natale DA, O'Donovan C, Redaschi N, Yeh LS (2004). UniProt: the universal protein knowledgebase. Nucleic Acids Res.

[B19] Altschul SF, Madden TL, Schaffer AA, Zhang J, Zhang Z, Miller W, Lipman DJ (1997). Gapped BLAST and PSI-BLAST: a new generation of protein database search programs. Nucleic Acids Res.

[B20] Graham MA, Smith KAT, Cannon SB, VandenBosch KA (2004). Computational Identification and Characterization of Novel Genes from Legumes. Plant Physiology.

[B21] Hansen NC, Schmitt MA, Anderson JE, Strock JS (2003). Soybean: Iron Deficiency of Soybean in the Upper Midwest and Associated Soil Properties. Agronomy Journal.

[B22] USDA A, National Genetic Resources Program (2004). Germplasm Resource Information Network – GRIN.

[B23] Connolly EL, Guerinot M (2002). Iron stress in plants. Genome biology.

[B24] Bae H, Kim MS, Sicher RC, Bae HJ, Bailey BA (2006). Necrosis and ethylene inducing peptide from Fusarium oxysporum induces a complex cascade of transcripts associated with signal transduction and cell death in Arabidopsis. Plant Physiol.

[B25] Branco-Price C, Kawaguchi R, Ferreira RB, Bailey-Serres J (2005). Genome-wide Analysis of Transcript Abundance and Translation in Arabidopsis Seedlings Subjected to Oxygen Deprivation. Annals of botany.

[B26] Cominelli E, Galblati M, Vavasseur A, Conti L, Sala T, Vuylsteke M, Leonhardt N, Dellaporta S, Tonelli C (2005). A Guard-Cell-Specific MYB Transcription Factor Regulates Stomatal Movements and Plant Drought Tolerance. Current Biology.

[B27] Gong Q, Li P, Ma S, Indu Ruspassara S, Bohnert HJ (2005). Salinity Stress Adaptation Competence in the Extremophile Thellungiella halophila in Comparison with its Relative Arabidopsis thaliana. Plant J.

[B28] He XJ, Mu RL, Cao WH, Zhang ZG, Zhang JS, Chen SY (2005). AtNAC2, a transcription factor downstream of ethylene and auxin signaling pathways is involved in salt stress response and latereal root development. The Plant Journal.

[B29] Kasukabe Y, He L, Nada K, Misawa S, Ihara I, Tachibana S (2004). Overexpression of Spermidine Synthase Enhances Tolerance to multiple Environmental Stresses and Up-Regulates the Expression of Various Stress Regulated Genes in Transgenic Arabidopsis thaliana. Plant Cell Physiology.

[B30] Rizhsky L, Davletova H, Liang H, Mittler R (2004). The Zinc Finger Protein Zat12 Is Required for Cytosolic Ascorbate Peroxidase 1 Expression during Oxidative Stress in Arabidopsis. The Journal of biological chemistry.

[B31] Wong CE, Li Y, Labbe AS, Guervara D, Nuin P, Whitty B, Xiaz C, Golding GB, Gray GR, Weretilnyk EA, Griffith M, Moffatt BA (2006). Transcriptional Profiling Implicates Novel Interactions between Abiotic Stress and Hormonal Responses in Thellungiella, a Close Relative of Arabidopsis. Plant Physiology.

[B32] Graham AM, Ramirez M, Valdes-Lopez O, Lara M, Tesfaye M, Vance CP, Hernandez G (2006). Identification of candidate phosphorus stress induced genes in Paseolus vulgaris L. through clustering analysis across several plant species. Functional Plant Biology.

[B33] Negishi T, Nakanishi H, Yazaki J, Kishimoto N, Fujii F, Shimbo K, Yamamoto K, Sakata K, Sasaki T, Kikuchi S, Mori S, Nishizawa NK (2002). cDNA microarray analysis of gene expression during Fe-deficiency stress in barley suggests that polar transport of vesicles is implicated in phytosiderophore secretion in Fe-deficient barley roots. The Plant Journal.

[B34] Misson J, Raghothama KG, Jain A, Jouhet J, Block MA, Bligny R, Ortet P, Creff A, Somerille S, Rolland N, Doumas P, Narcy P, Herrera-Estrella L, Nussaume L, Thibaud MC (2005). A genome-wide transcriptional analysis using Arabidopsis thaliana Affymetrix gene chips determined plant responses to phosphate deprivation. Proceedings of the National Academy of Sciences of the United States of America.

[B35] Fujimoto SY, Ohta M, Usui A, Shinshi H, Ohme-Takagi M (2000). Arabidopsis Ethylene-Responsive Element Binding Factors Act as Transcriptional Activators or Repressors of GCC Box-Mediated Gene Expression. The Plant Cell.

[B36] Schikora A, Schmidt W (2001). Iron stress-induced changes in root epidermal cell fate are regulated independently from physiological responses to low iron availability. Plant Physiol.

[B37] Waters BM, Chu HH, Didonato RJ, Roberts LA, Eisley RB, Lahner B, Salt DE, Walker EL (2006). Mutations in Arabidopsis yellow stripe-like1 and yellow stripe-like3 reveal their roles in metal ion homeostasis and loading of metal ions in seeds. Plant Physiol.

[B38] Schmidt W, Tittel J, Schikora A (2000). Role of hormones in the induction of iron deficiency responses in Arabidopsis roots. Plant Physiol.

[B39] Ohme-Takagi M, Suzuki K, Shinshi H (2000). Regulation of Ethylene-Induced Transcription of Defense Genes. Plant Cell Physiology.

[B40] Wang KLC, Li H, Ecker JR (2002). Ethylene Biosynthesis and Signaling Networks. The Plant Cell.

[B41] Haas C, Lohrmann J, Albrecht V, Sweere U, Hummel F, Yoo SD, Hwang I, Zhu T, Schafer E, Kudla J, Harter K (2004). The response regulator 2 mediates ethylene signaling and hormone signal integration in Arabidopsis. The EMBO Journal.

[B42] Bleecker AB, Kende H (2000). Ethylene: a gaseous signal molecule in plants. Annual review of cell and developmental biology.

[B43] Sperotto RA, Riachenevsky FK, Fett JP (2007). Iron deficiency in rice shoots: identification of novel induced genes using RDA and possible relation to leaf senescence. Plant cell reports.

[B44] Noguchi A, Saito A, Homma Y, Nakao M, Sasaki N, Nishino T, Takahashi S, Nakayama T (2007). A UDP-Glucose:Isoflavone 7-O-Glucosyltransferase from the Roots of Soybean (Glycine max) Seedlings: PURIFICATION, GENE CLONING, PHYLOGENETICS, AND AN IMPLICATION FOR AN ALTERNATIVE STRATEGY OF ENZYME CATALYSIS. The Journal of biological chemistry.

[B45] Plou FJ, Martin MT, Gomez de Segura A, Alcalde M, Ballesteros A (2002). Gluccosyltransferases acting on starch or sucrose for the synthesis of oligosaccharides. Canadian Journal of Chemistry.

[B46] Parodi AJ, Mendelzon DH, Lederkremer GZ, Martin-Barrientos J (1984). Evidence that transient glucosylation of protein-linked Man9GlcNAc2, Man8GlcNAc2, and Man7GlcNAc2 occurs in rat liver and Phaseolus vulgaris cells. The Journal of biological chemistry.

[B47] Tipping AJ, McPherson MJ (1995). Cloning and molecular analysis of the pea seedling copper amine oxidase. The Journal of biological chemistry.

[B48] Sun B, Jing Y, Chen K, Song L, Chen F, Zhang L (2007). Protective effect of nitric oxide on iron deficiency-induced oxidative stress in maize (Zea mays). Journal of plant physiology.

[B49] Zaharieva TB, Abadia J (2003). Iron deficiency enhances the levels of ascorbate, glutathione, and related enzymes in sugar beet roots. Protoplasma.

[B50] Ranieri A, Castagna A, Baldan B, Soldatini GF (2001). Iron deficiency differently affects peroxidase isoforms in sunflower. Journal of experimental botany.

[B51] Rose TL, Bonneau L, C. D, Marty-Mazars D (2006). Starvation – induced expression of autophagy-related genes in Arabidopsis. Biol Cell.

[B52] Alignan M, Hewezi T, Petitprez M, Dechamp-Guillaume G, Gentzbitten L (2006). A cDNA microarray approach to decipher sunflower (Helianthus annuus) responses to the necrotrophic fungusPhoma macdonaldii. New Phytologist.

[B53] Goritschnig S, Zhang Y, Li X (2007). The ubiquitin pathway is required for innate immunity in Arabidopsis. The Plant Journal.

[B54] Thimm O, Essigmann B, Kloska S, Altmann T, Buckhout TJ (2001). Response of Arabidopsis to Iron Deficiency Stress as Revealed by Microarray Analysis. Plant Physiology.

[B55] Garbarino JE, Oosumi T, Belknap WR (1995). Isolation of a polyubiquitin promoter and its expression in transgenic potato plants. Plant Physiology.

[B56] Hudson BP, Martinez-Yamout MA, Dyson HJ, Wright PE (2004). Recognition of the mRNA AU-rich element by the zinc finger domain of TIS11d. Nature structural & molecular biology.

[B57] Connolly EL, Fett JP, Guerinot ML (2002). Expression of the IRT1 metal transporter is controlled by metals at the levels of transcript and protein accumulation. The Plant cell.

[B58] Brumbarova T, Bauer P (2004). Iron-Mediated Control of the Basic Helix-Loop-Helix Protein FER, a Reulator of Iron Uptake in Tomato. Plant Physiology.

[B59] Aravind L, Koonin EV (2001). The DNA-repair protein AlkB, EGL-9, and leprecan define new families of 2-oxoglutarate- and iron-dependent dioxygenases. Genome biology.

[B60] Rietz S, Holk A, Scherer GF (2004). Expression of the patatin-related phospholipase A gene AtPLA IIA in Arabidopsis thaliana is up-regulated by salicylic acid, wounding, ethylene, and iron and phosphate deficiency. Planta.

[B61] Agarwal P, Arora R, Ray S, Singh AK, Singh VP, Takatsuji H, Kapoor S, Tyagi AK (2007). Genome-wide identification of C(2)H (2) zinc-finger gene family in rice and their phylogeny and expression analysis. Plant molecular biology.

[B62] Ray S, Agarwal P, Arora R, Kapoor S, Tyagi AK (2007). Expression analysis of calcium-dependent protein kinase gene family during reproductive development and abiotic stress conditions in rice (Oryza sativa L. ssp. indica). Mol Genet Genomics.

[B63] Fehr WR, Caviness CE (1977). Stages of Soybean Development, Special Report 80.

[B64] Barrett T, Troup DB, Wilhite SE, Ledoux P, Rudnev D, Evangelista C, Kim IF, Soboleva A, Tomashevsky M, Edgar R (2007). NCBI GEO: mining tens of millions of expression profiles–database and tools update. Nucleic Acids Res.

[B65] Edgar R, Domrachev M, Lash AE (2002). Gene Expression Omnibus: NCBI gene expression and hybridization array data repository. Nucleic Acids Res.

[B66] Schmittgen TD, Zakrajsek BA, Mills AG, Gorn V, Singer MJ, Reed MW (2000). Quantitative Reverse Transcription-Polymerase Chain Reaction to Study mRNA Decay: Comparison of Endpoint and Real-Time Methods. Analytical Biochemistry.

[B67] Gong Q, Li P, Ma S, Indu Rupassara S, Bohnert HJ (2005). Salinity stress adaptation competence in the extremophile Thellungiella halophila in comparison with its relative Arabidopsis thaliana. Plant J.

[B68] Kasukabe Y, He L, Nada K, Misawa S, Ihara I, Tachibana S (2004). Overexpression of spermidine synthase enhances tolerance to multiple environmental stresses and up-regulates the expression of various stress-regulated genes in transgenic Arabidopsis thaliana. Plant & cell physiology.

